# Changes in bone turnover markers and bone modulators during abatacept treatment

**DOI:** 10.1038/s41598-023-44374-2

**Published:** 2023-10-11

**Authors:** Giovanni Adami, Giovanni Orsolini, Maurizio Rossini, Elisa Pedrollo, Anna Fratucello, Angelo Fassio, Ombretta Viapiana, Stefano Milleri, Elena Fracassi, Riccardo Bixio, Davide Gatti

**Affiliations:** 1 Rheumatology Unit, Azienda Ospedaliera Universitaria Integrata di Verona, University of Verona, Pz Scuro 10, 37134 Verona, Italy; 2 Research Unit, Azienda Ospedaliera Universitaria Integrata di Verona, Verona, Italy; 3 Centro Ricerche Cliniche (CRC), Azienda Ospedaliera Universitaria Integrata di Verona, Verona, Italy

**Keywords:** Immunology, Inflammation, Osteoimmunology

## Abstract

Rheumatoid arthritis (RA) causes bone loss, only partly related to inflammation. The impact of RA treatments on bone metabolism and their ability to mitigate bone loss remains uncertain. The primary goal of our study was to examine the influence of abatacept on serum levels of markers and regulators involved in bone turnover. Secondary objectives included evaluating changes in bone mineral density (BMD), bone health parameters, erosions, and exploring potential correlations among these parameters. We conducted a prospective observational study on patients with active seropositive RA failure to biological disease modifying anti-rheumatic drugs initiating treatment with abatacept. We measured at baseline and after 1, 2, 3, 6, 9 and 12 months: serum bone turnover markers (CTX, P1nP, B-ALP), bone modulators (Dkk-1, sclerostin, vitamin D, PTH, OPG and RANKL), BMD and radiographic parameters (modified Sharp van der Heijde score [mSvdH], bone health index [BHI] and metacarpal index [MCI]). Disease activity and glucocorticoid intake was monitored. 33 patients were enrolled in the study. We found a significant increase in markers of bone formation (B-ALP and P1nP) from baseline to M6 and M12. PTH increased significantly at M6 but not at M12. All other bone markers and modulators did not change. We found a significant decrease in BHI and MCI from baseline to M12 (median difference − 0.17 95% CI − 0.42 to − 0.10, *p* 0.001 and − 0.09 95% CI − 0.23 to − 0.07, respectively). BMD at femoral neck transitorily decreased at M6 (mean difference − 0.019 g/cm^2^ 95% CI − 0.036 to − 0.001 *p* 0.04). BMD at total hip, lumbar spine and mSvdH score did not change significantly. P1nP delta at M12 correlated with delta mSvdH. Treatment with abatacept was associated with a significant increase in bone formation markers. The secondary and transient increase in PTH serum levels may be responsible of the transitory bone loss.

## Introduction

Rheumatoid arthritis (RA) is a chronic autoimmune disease characterized by persistent inflammation, which leads to joint damage and functional limitations^1^. In addition to its impact on the joints, RA is associated with various systemic complications, including osteoporosis and an increased susceptibility to fractures^2^. Although factors like glucocorticoid use and comorbidities contribute to bone loss in RA patients, there are other metabolic factors are likely to play a significant role^3,4^.

Bone remodeling is a complex process involving the continuous cycle of bone formation and resorption, which is tightly regulated by numerous signaling pathways. Among these pathways, the Wnt signaling pathway has emerged as a crucial regulator of joint and bone remodeling, especially in the context of RA^5,6^. Specifically, molecules inhibiting the Wnt pathway, such as sclerostin and dickkopf-1 (Dkk1), have been found to be abnormally expressed in RA patients compared to both healthy individuals and those with other inflammatory rheumatic musculoskeletal diseases^7–9^. However, it remains uncertain whether disease-modifying antirheumatic drugs (DMARDs) can directly modulate the metabolic profile of RA, specifically targeting bone cells and bone metabolism.

Abatacept, a fusion protein of cytotoxic T lymphocyte- associated antigen-4 and immunoglobulin G1 that selectively modulates the CD80/CD86:CD28 costimulatory signal required for full T-cell activation, has demonstrated efficacy in controlling inflammation related to RA^10^. While previous studies have explored the effects of targeted therapies, particularly tumor necrosis factor (TNF) inhibitors, on bone remodeling in RA, limited research has investigated the impact of abatacept on bone remodeling and bone modulators^11–14^.

The primary objective of our study was to investigate the influence of abatacept on bone turnover markers, bone modulators and bone health overall in patients with RA. Understanding the effects of abatacept on bone in RA patients is crucial for optimizing treatment strategies and mitigating the risk of osteoporosis and fractures. By elucidating the direct effects of abatacept on bone health, our study aims to contribute to a comprehensive understanding of the metabolic alterations associated with RA and the potential therapeutic interventions. This knowledge has the potential to enhance the management of RA and improve patient outcomes.

## Material and methods

We conducted an observational study on patients with active seropositive (either rheumatoid factor [RF] or Anti-citrullinated protein antibodies [ACPAs] positive or both) RA who had not responded to targeted synthetic or biologic DMARDs (tsDMARDs or bDMARDs) on stable dose of glucocorticoids (prednisone equivalent ≤ 5 mg/day) for at least 3 months. Patients initiated weekly subcutaneous dose of abatacept 125 mg. Throughout a 12-month period, the patients were regularly monitored and underwent control visits and study procedures at specific time points: baseline (B), month 1 (M1), month 2 (M2), month 3 (M3), month 6 (M6), month 9 (M9), and month 12 (M12). Modifying the dose of conventional synthetic DMARDs (csDMARDs) and administering a short course of glucocorticoids at doses higher than 5 mg/day (prednisone equivalent) for less than 15 days were permitted; however, any changes made were recorded in detail. In accordance with the study protocol, investigators aimed to keep the DMARDs and glucocorticoid doses as stable as possible while ensuring proper treatment for the patients, hence allowing for glucocorticoid tapering if necessary.

### Inclusion and exclusion criteria

The inclusion criteria for this study were as follows: participants aged 18 years or older, provide signed informed consent, with diagnosis of RA and fulfilling the ACR/EULAR 2010 criteria, seropositive to either ACPA or RF, experience disease onset within the past 3 years, require abatacept treatment due to failure or intolerance to tsDMARDs or bDMARDs, maintain a stable glucocorticoid dose at or below 5 mg/day of prednisone equivalent for at least 3 months before enrollment, undergo a washout period of at least 3 half-lives of the previous bDMARDs tsDMARDs, and have a washout period of at least 3 months.

The exclusion criteria were as follows: presence of any other rheumatic diagnosis beside RA, presence of bone diseases other than osteoporosis (such as Paget's disease of the bone), severe liver or kidney disease (estimated glomerular filtration rate < 30 mL/min or Child–Pugh grade B or C), uncontrolled endocrine disease, contraindication to abatacept based on the label instructions, recent fracture (6 months prior to enrollment), concurrent use of bisphosphonates (oral bisphosphonates for up to 12 months and zoledronic acid for up to 24 months), use of strontium ranelate, teriparatide, selective estrogen receptor modulators , or denosumab, intra-articular injections at the metacarpophalangeal joints within the 3 months prior to the study (to limit the potential impact of steroid on hands x-ray images), and pregnancy or breastfeeding status.

We enrolled consecutive out-patients who visited the clinic for their regular checkups. Patients were included in the study based on their availability and willingness to participate. No specific selection criteria (other than inclusion/exclusion criteria) were applied. Patients were enrolled over a 6-month period, from June 2021 to December 2021.

All clinical evaluations were performed by a single expert rheumatologist (GO) to minimize variability in measurements.

### Sample size

This was an exploratory biomarker development study, the sample size of 30 patients was based on clinical judgement and practical considerations and not on formal statistical reasoning.

### Study procedures

Prior to enrollment in the study, patients underwent screening to determine if they met the inclusion and exclusion criteria. If they met the criteria, they attended a baseline visit. At each visit (baseline, M1, M2, M3, M6, M9, and M12), clinical and safety assessments were conducted. The current treatments for RA (glucocorticoid and conventional DMARDs doses) and other medications were reviewed, and the Disease Activity Score based on 28 joints C-reactive protein (DAS28-CRP) was calculated. Bone mineral density (BMD) measurements were taken at the femoral neck and lumbar spine (L1-L4) using dual-energy X-ray absorptiometry (DXA) with the QDR Hologic Delphi machine at baseline, M6, and M12. The variation coefficient for the vertebral site was 1%, while it was 1.2% for the femoral neck. At baseline and M12, plain X-rays of the hands and feet were performed. The modified Sharp/van der Heijde score (mSvdH score) was calculated by two independent expert readers. The hand X-ray images in DICOM format were analyzed using Bonexpert for Adults software by Visiana version 2.3.0.4. The software provided data on the Bone Health Index (BHI) and metacarpal index (MCI), which are indicators of cortical thickness based on the width and length of the three middle metacarpal bones. The average values (right and left) of BHI and MCI were calculated. Blood samples were collected in the morning after fasting at baseline, M1, M2, M3, M6, and M12. The serum samples were aliquoted and stored at − 80 °C until they were assayed for various markers. These markers included C-terminal telopeptide of type I collagen (CTX, a marker of bone resorption), procollagen I intact N-terminal peptide (P1NP, a marker of bone formation), bone alkaline phosphatase (B-ALP, a marker of bone formation), Dkk1 (a Wnt inhibitor), sclerostin (a Wnt inhibitor), 25OH-vitamin D (25OHVitD), parathyroid hormone (PTH), osteoprotegerin (OPG), and receptor activator of nuclear factor kappa-Β ligand (RANKL). The measurements of CTX, P1NP, and B-ALP were performed using the IDS-ISYS Multi-Discipline Automated Analyzer based on chemiluminescence technology. The intra-assay coefficients of variation were 3.0% for P1NP, 2% for CTX, and 4% for B-ALP. Serum Dkk1 and sclerostin were measured using ELISA kits, with sensitivities of 0.89 and 8.9 pmol/L, respectively, and intra-assay coefficients of variation of 7.8% and 5.6%, respectively. The inter-assay variabilities were 8.2% and 6.9% for Dkk1 and sclerostin, respectively. PTH was measured using ELISA with an intra-assay variability of 6% and an inter-assay variability of 7%. 25OHVitD was measured using the LIAISON® 25OHVitD assay, with an intra-assay variability of 8% and an inter-assay variability of 12%. Serum RANKL and OPG were measured using ELISA kits, with intra-assay coefficients of variation of 8.1% and 6.3%, respectively. The inter-assay variabilities were 9.1% and 7.2% for RANKL and OPG, respectively. To minimize inter-assay variability, all samples were measured in a single batch.

### Statistical analysis

Normality was tested with both D'Agostino-Pearson test and the Shapiro–Wilk test. In addition, frequency was visually inspected to confirm or exclude normal distribution.

Group comparisons were performed with t-student and Mann–Whitney U tests (for normally and non-normally distributed continuous variables, respectively). Categorical variables were compared with χ^2^ test. Associations between continuous variables were tested using Pearson correlation coefficients. To account for multiplicity, we used the False Discovery Rate (FDR) approach with the two-stage step-up method of Benjamini, Krieger and Yekutieli (Q value 5% of FDR).

We analyzed BMD, serum markers and clinical measures changes with mixed-effect model analysis for repeated measures. If there are missing values in the data, repeated measures analysis of variance (ANOVA) cannot be used. Instead, we utilized GraphPad Prism 9.5.1 to analyze the data by fitting a mixed model that employs a compound symmetry covariance matrix, with a restricted maximum likelihood approach. It's worth noting that in the absence of missing values, this approach provides the same p-values and multiple comparisons tests as the repeated measures ANOVA. We adjusted p-values in multiple comparisons using the Tukey’s procedure. SvdH score, BHI and MCI differences were tested with Wilcoxon Signed-Ranks Test for paired groups.

All differences were considered significant when *p* value was inferior to 0.05.

All statistical analyses were performed using SPSS Version 26 (SPSS, Inc., Chicago, IL, USA) and GraphPad Prism version 9.5.1 (GraphPad Software, San Diego, CA, USA). This study was approved by the University of Verona ethic committee (prot. 1758CESC).

### Ethics approval and consent to participate

The study was conducted according to the protocol 1758CESC approved by our local Ethics Committee, in accordance with the 1964 Helsinki declaration and its later amendments or comparable ethical standards. Informed consent was collected for each participant.

## Results

### Patients characteristics

Thirty-three patients (86.7% women) that satisfied inclusion criteria were consecutively enrolled in the study. Mean age was 59.0 years (SD 13.1), mean disease duration was 1.2 years (SD 0.4), all patients, as per inclusion criteria, had seropositive RA. All patients were concomitantly treated with csDMARD (26 with methotrexate, 6 with leflunomide and 3 with sulfasalazine). Patients characteristics are given in Table 1. Complete data analysis was available for 21 patients in whom the baseline characteristics did not differ significantly from the overall population (data not shown). Disease activity significantly decreased from baseline (mean DAS28-CRP 4.3, SD 0.9) to M12 (mean DAS28-CRP 2.3, SD 1.1), *p* for linear trend < 0.0001 (Figure S1 supplementary materials).

**Table 1:** Characteristics of the study population.

Characteristics	n = 33
Sex, female (%)	28 (85%)
Age, years (SD)	59.0 (13.1)
Menopause, n (% of 28 women)	23 (82%)
Weight, kg (SD)	76.3 (14.7)
Height, cm (SD)	165 (7)
ACPA positive, n (%)	33 (100%)
RF positive, n (%)	26 (78.8%)
Disease duration, years (SD)	1.2 (0.4)
Baseline swollen joints, n (IQR)	5 (4)
Baseline tender joints, n (IQR)	5 (5)
Baseline PGA (IQR)	6 (2)
Baseline PhGA (IQR)	7 (2)
Baseline CRP serum levels, mg/L (IQR)	3 (6.5)
Baseline DAS28-CRP (SD)	4.3 (0.9)
Month 12 DAS28-CRP (SD)	
Baseline mSvdH score (IQR)	25 (46.25)
Treatment with cDMARD, n (%)	
Methotrexate	26 (79%)
Leflunomide	6 (18%)
Sulfasalazine	3 (9%)
Vitamin D supplements taking, n (%)	26 (79%)
GC taking, n (%)	20 (61%)
GC daily dose at enrollment, mg (IQR)	5 (5)
GC cumulative dose prior to enrollment, mg (IQR)	1291 (2196)
GC cumulative dose during study period, mg (IQR)	300 (784.5)
GC daily dose at month 12, mg (IQR)	0 (3.25)
ACPA titer, IU (IQR)	853 (1584)
RF titer, IU (IQR)	60 (130)
Smoking habit, n (%)	
No	18 (55%)
Yes	8 (24%)
Former	7 (21%)
Charlson Comorbidity Index (IQR)	2 (2.25)

*SD* standard deviation; *ACPA* anti-citrullinated proteins antibodies; *RF* rheumatoid factor; *IQR* interquartile range; *PGA* patient global assessment; *PhGA* physician global assessment; *CRP* c-reactive protein; *DAS28* disease activity score 28-joints; *csDMARD* conventional synthetic disease modifying anti-rheumatic drugs; *GC* glucocorticoids*.*

### Bone turnover markers

**Figure 1:**
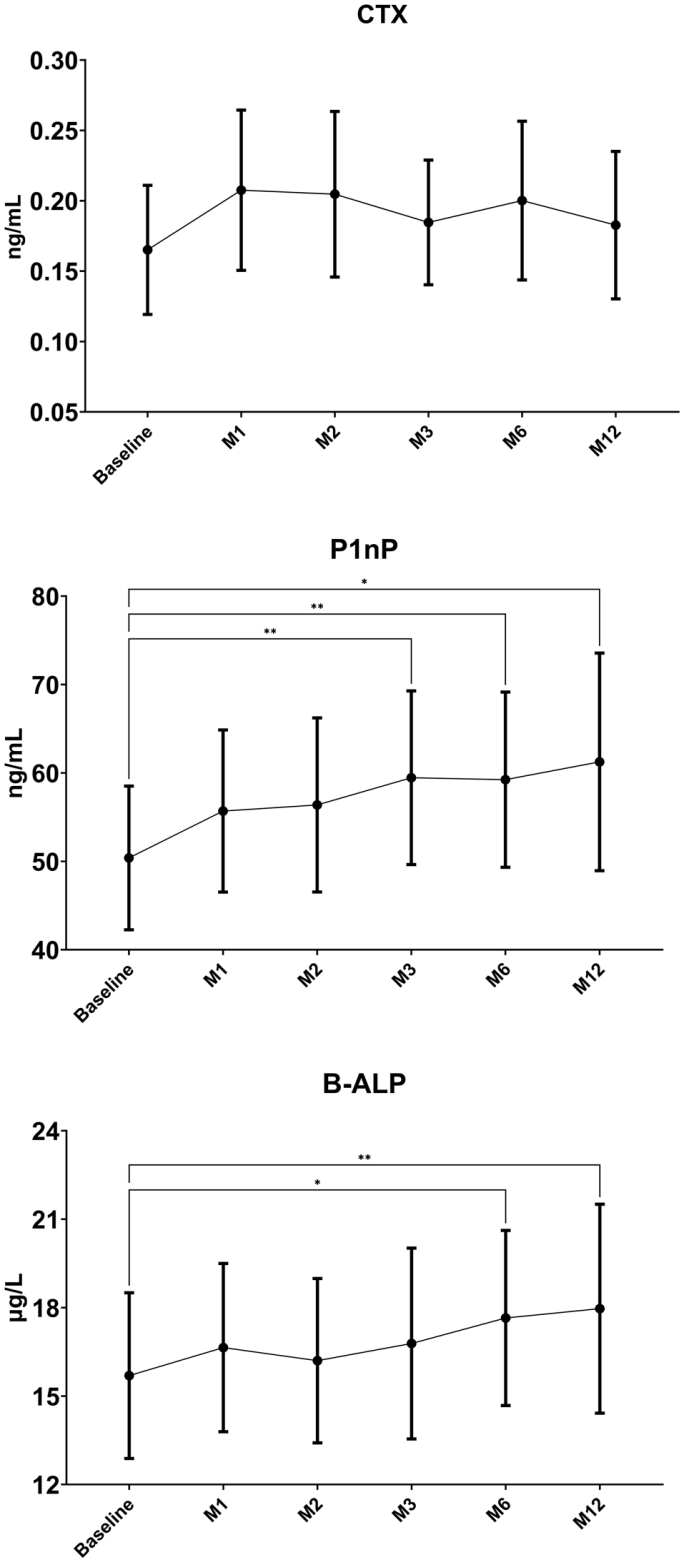
Changes in bone turnover markers (BTMs) in the study period. Changes were tested with mixed-effect model analysis for repeated measures (MMRM), *p* values are adjusted for multiple comparisons using the Tukey’s procedure.

**Figure 2:**
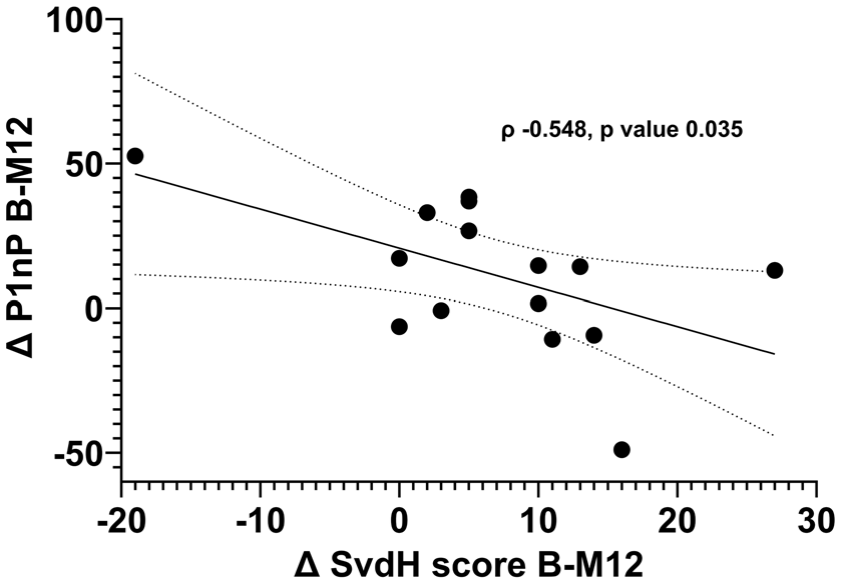
Negative correlation between delta P1nP between baseline and M12 and delta Sharp van der Heijde (SvdH) between baseline and M12.

In Fig. 1 are shown the changes of bone turnover markers in the study period. P1nP significantly increased at M3 (mean difference + 9.1 pg/mL 95% CI 2.8 to 15.3 *p* value 0.006). B-ALP and P1nP significantly increased at M6 (mean difference + 1.9 µg/L 95% CI 0.3 to 3.6 *p* value 0.02 and + 8.8 pg/mL 95% CI 3.2–14.5 *p* value 0.003, respectively) and M12 (mean difference + 2.3 µg/L 95% CI 0.7 to 3.9 p value 0.007 and + 10.9 pg/mL 95% CI 0.8–20.9 *p* value 0.03, respectively). CTX did not change. P1nP levels were significantly lower in glucocorticoid taking subjects compared to non-taking subjects (45.5 [SD 24.7] vs. 58.0 [SD 16.0], respectively, *p* 0.048). Other markers did not differ significantly between glucocorticoid users and non-users at baseline. P1nP and B-ALP levels increase was less pronounced in glucocorticoids users compared to non-users (Figure S2 and figure S3 supplementary materials). We did not find any significant difference in bone markers during the study period when stratified for glucocorticoid tapering (patients tapering glucocorticoids vs patients on stable glucocorticoid dose during the study). We also found that P1nP reduction was significant only in patients with DAS28-CRP > 4.0 at baseline, whereas was somewhat stable in patient with DAS28-CRP ≤ 4.0 (DAS28-CRP threshold based on median value). We found a significant, negative, correlation between baseline—M12 delta P1nP and baseline—M12 delta mSvdH (Fig. 2). Results were corrected for multiplicity.

### Bone metabolism modulators

**Figure 3:**
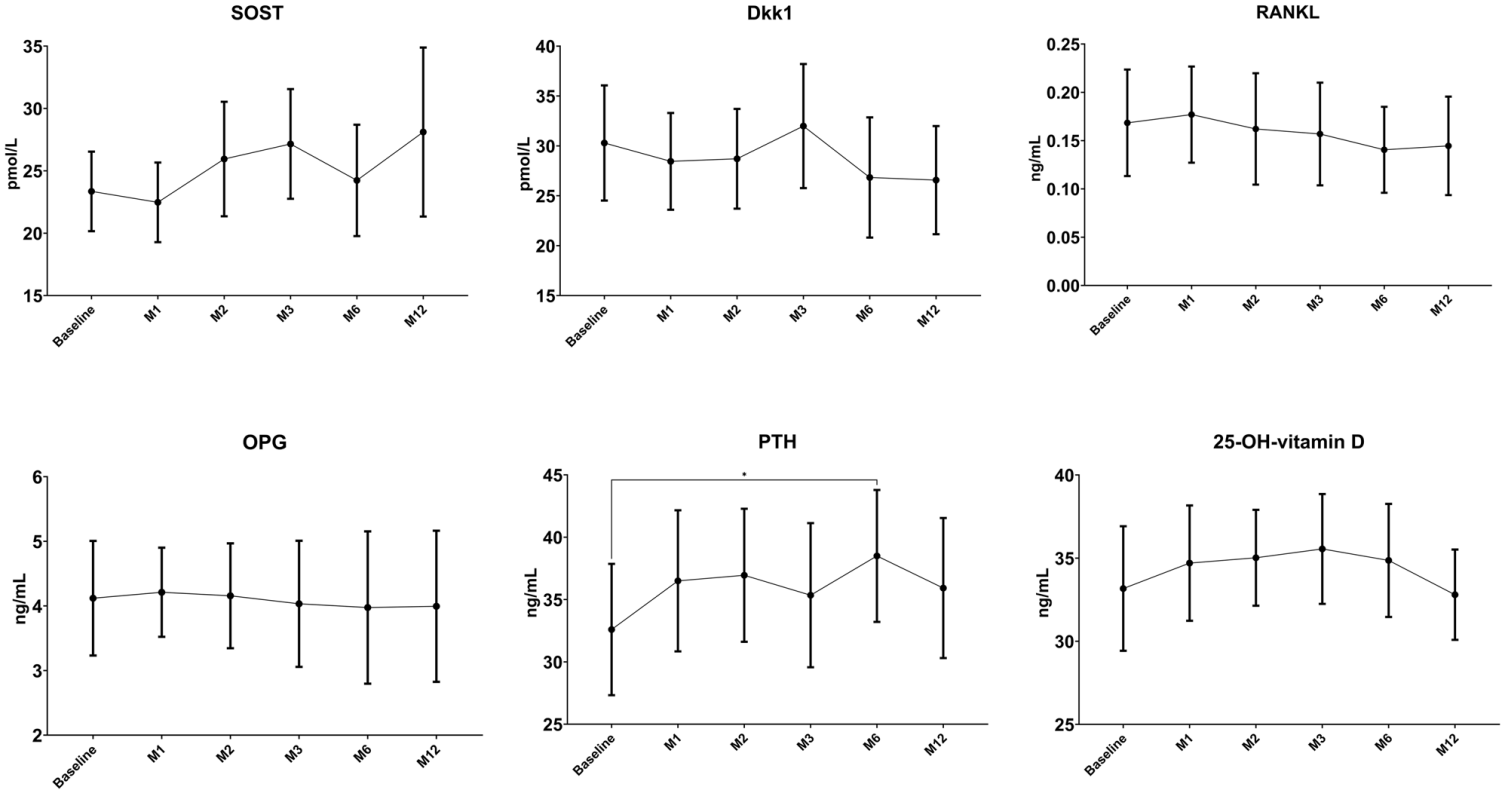
Changes in serum bone modulators in the study period. Changes were tested with mixed-effect model analysis for repeated measures (MMRM), *p* values are adjusted for multiple comparisons using the Tukey’s procedure.

In Fig. 3 are shown the changes in Dkk1, sclerostin, RANKL, OPG, vitamin D and PTH serum levels in the study period. Among bone metabolism modulators PTH increased significantly at M6 (+ 5.9 pg/mL 95% CI 1.4 to 10.4 *p* value 0.01) but not at M12. All other bone modulators did not change.

### Bone mineral density

**Figure 4:**
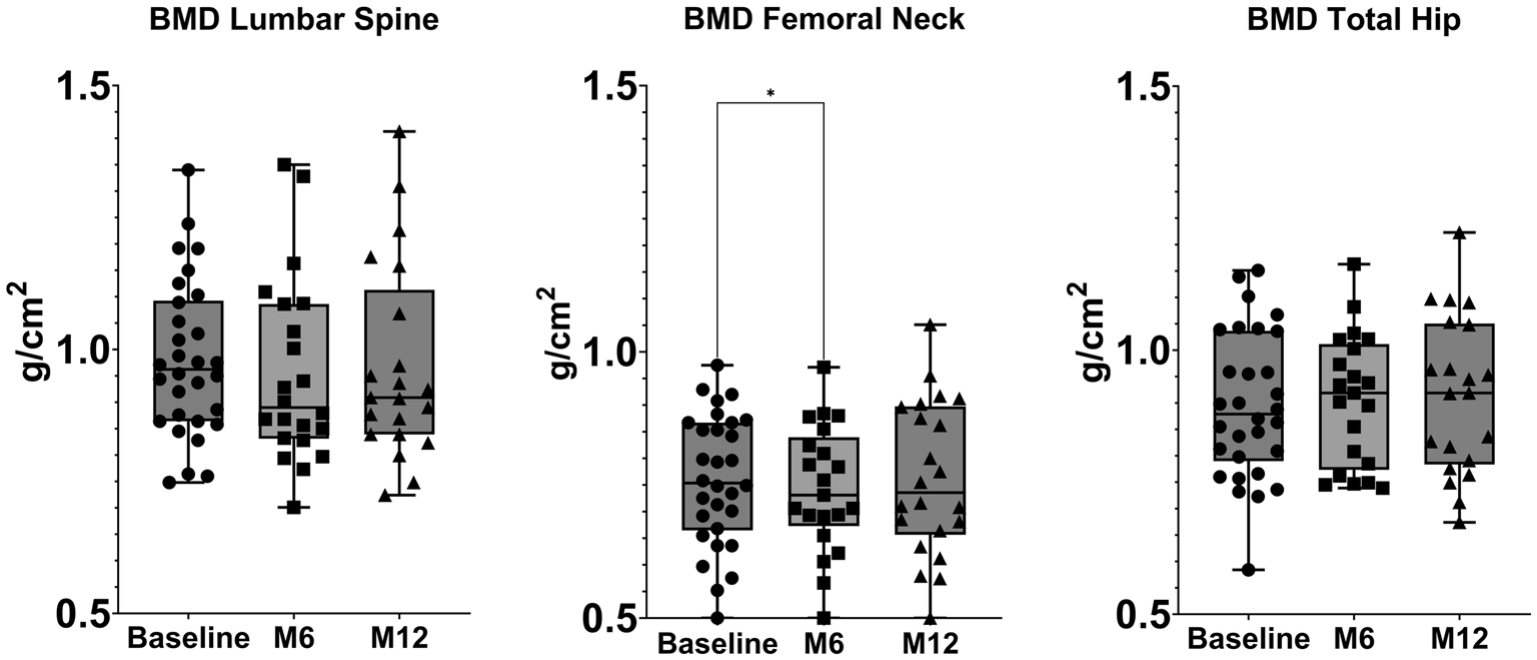
Changes in bone mineral density (BMD) at lumbar spine, total hip and femoral neck in the lower panel. Changes were tested with mixed-effect model analysis for repeated measures (MMRM), p-values are adjusted for multiple comparisons using the Tukey’s procedure.

Mean BMD levels at baseline were: 0.981 (SD 0.143), 0.759 (SD 0.120) and 0.895 (SD 0.135) at lumbar spine, femoral neck and total hip, respectively. BMD (both at lumbar spine, femoral neck and total hip) at baseline did not correlate with any of the patients’ characteristics or baseline markers serum levels (p adjusted for multiplicity NS). Femoral neck BMD decreased significantly between baseline and M6 (mean difference -0.019 g/cm^2^ 95% CI − 0.036 to − 0.001 *p* 0.04). BMD at other sites or other time points did not change significantly throughout the study period. BMD changes are shown in Fig. 4. Since BMD levels are generally higher in men, we conducted a sub-analysis excluding male subjects and we did not find any significant difference in results (data not shown).

### mSvdH score, BHI and MCI

**Figure 5:**
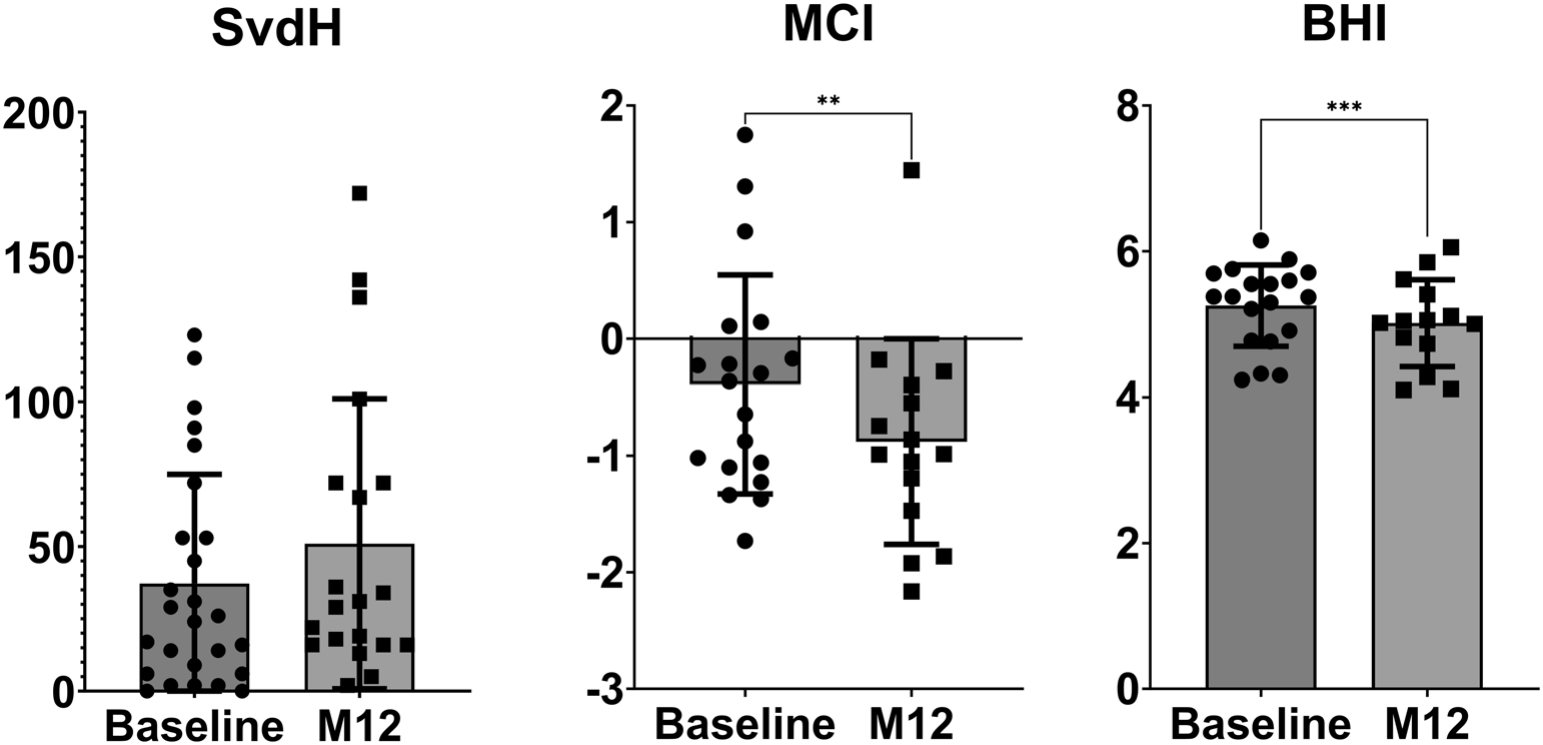
Changes in Sharp van der Heijde (SvdH) score, metacarpal index (MCI) and bone health index (BHI). Changes were tested with Wilcoxon signed-ranks test.

mSvdH score did not change form baseline to M12 (median score 25, IQR 8–51 and 25.5, IQR 16–59 at baseline and M12 respectively, p NS). BHI and MCI significantly decreased from baseline to M12 (median difference − 0.17 95% CI − 0.42 to − 0.10, *p* 0.001 and − 0.09 95% CI − 0.23 to − 0.07, respectively). SvdH score, BHI and MCI changes are depicted in Fig. 5.

## Discussion

The present study aimed to investigate the impact of abatacept, a CD80–CD86 modulator, on bone turnover markers, bone modulators, and bone health in patients with RA. The findings may shed light on the potential effect of abatacept on bone metabolism and provide insights into the underlying mechanisms and implications for optimizing treatment strategies.

We found a significant increase in bone formation markers, specifically P1NP and B-ALP, following abatacept treatment. P1NP, a marker of bone formation, showed a significant increase at month 3, while both P1NP and B-ALP exhibited significant increases at months 6 and 12. These findings suggest that abatacept may enhance bone neoformation in patients with RA. However, due to the observational nature of the study, we cannot imply causal relationship. Nonetheless our result is particularly relevant in the context of RA, where chronic inflammation has been shown to greatly impact osteoblasts functionality and to disrupt the balance between bone formation and resorption, leading to bone loss^2,3,6^. Interestingly, Roser-Page and colleagues showed that inhibition of CD28 significantly increase bone formation in a mice model of arthritis^15^. Of note, we did find a correlation between delta P1nP at M12 and delta mSvdH score at M12 further highlighting the benefits of increasing bone formation on radiographic progression of RA. Again, however, we cannot make definitive assumption about causal relationship between P1nP increase and bone erosions from our results.

It is noteworthy that the transient increase in PTH levels at month 6 may have counteracted the positive effects of increased bone formation in a sort of para-physiologic feedback. PTH is a key regulator of calcium and phosphate metabolism, and it plays a crucial role in maintaining bone homeostasis^16^. In response to low serum calcium levels, PTH stimulates bone resorption to release calcium into the bloodstream^17^. Notably, studies on PTH physiology have demonstrated a sigmoidal relationship between serum ionized calcium and PTH levels^18^. This relationship indicates that even PTH levels within the normal range can impact calcium balance and bone resorption. Specifically, the inflection point of this sigmoidal curve falls within the normal PTH range, approximately around 35 pg/mL. When PTH levels exceed this threshold, a negative calcium balance occurs, with calcium moving from the bone to the extracellular compartment. In our study, we observed a significant increase in PTH levels from 32.59 to 38.50 pg/mL, which crossed this critical threshold. The observed increase in PTH levels may have resulted from the transient imbalance caused by increased bone formation. This transient increase in PTH could have contributed to the bone loss (i.e., BHI, MCI and femoral neck BMD), particularly in the early phases of abatacept treatment. Similarly, we previously showed that TNF inhibition, in RA patients, led to an increase of PTH^19^. In contrast to our results, Tada and colleagues previously showed that abatacept was associated with a significant increase in femoral neck BMD^11^. However, they assessed BMD only at 12 months of therapy, possibly missing the early decrease in BMD that is typically seen in patients with a transitory increase in PTH serum levels. For example, teriparatide treatment is associated with a significant, but arguably temporary, decrease in bone density (i.e., femoral neck and distal radius), which is secondary to cortical enlargement and increase porosity^20–22^. However, later in the treatment course (12–24 months), especially when an anti-resorptive drug is subsequently administered, bone remodeling cavities are mineralized, and cortical bone density improves. Nonetheless, in RA patients this early decrease in cortical BMD might be detrimental and might predispose to erosions or attenuate the anti-erosive effects of DMARDs^23^. Interestingly, denosumab, which is particularly effective on cortical bone^24^, outperformed teriparatide in terms of bone erosion prevention and treatment^25–29^. Preclinical studies showed the potential for a potent antiresorptive effect of abatacept in RA^30–32^. We did not confirm such effect, which might have been negatively influenced by the increase in PTH. Nonetheless, future studies applying high resolution peripheral quantitative computed tomography might help disentangle such aspect.

To mitigate the potential negative impact of the early increase in PTH, we might speculate that increasing vitamin D levels may be beneficial. Adequate vitamin D levels are crucial for optimal bone health, and supplementation may help counteract the potential adverse effects of increased PTH levels on bone^33^. Moreover, vitamin D acts synergistically with PTH to regulate bone remodeling. However, it should be noted that the study patients had stable and normal levels of vitamin D throughout the study, and we did not directly investigate the impact of vitamin D supplementation in conjunction with abatacept treatment, and this remains a speculative suggestion based on observed changes in PTH levels. It has been shown that vitamin D levels might impact on disease activity, pain, and inflammation in RA^34–36^. Recent evidence suggests that daily vitamin D supplements reduced the incidence of autoimmune diseases, including RA^37^.

We also focused our study on bone modulators’. The measured bone modulators, including Dkk1, sclerostin, RANKL, and OPG, play critical roles in regulating bone remodeling and Wnt signaling pathways, especially in RA^3,6,38,39^. Interestingly, Kawashiri and colleagues reported that the baseline serum levels of Dkk1 were negative predictors of clinical response to abatacept, making this marker of particularly attractive from practical point of view^39^. Indeed, Dkk1 was consistently found elevated in RA patients in the literature^5,8,9^. However, we did not find any significant change in Dkk1 serum levels or other bone modulators. This might be, again, explained by the transient increase in PTH serum levels. We previously show that PTH is the major determinant of Dkk1 serum levels in various conditions, including RA^8,19,40,41^. The lack of significant changes in these markers might also suggest that abatacept may primarily influence bone remodeling through other mechanisms, rather than directly modulating the expression of these specific bone modulators. Another possible mechanism explaining the increase in bone formation might reside in decreasing glucocorticoid dose. In fact, glucocorticoids have been widely demonstrated to have a significant impact on bone health^42^, especially acting on osteoblast function^4,43,44^. Indeed, we found that P1nP levels were lower on average in patients taking glucocorticoids at baseline. Interestingly, P1nP and ALP increased earlier and more markedly in patients that did not take glucocorticoids at baseline. This result might be explained by the general suppression on bone formation exerted by glucocorticoids that might have blunted the possible positive effect of abatacept. However, contrasting with this hypothesis we did not find any significant difference in terms of bone anabolic markers in patients that tapered glucocorticoids or those who maintained a stable dose during the study. However, glucocorticoid dose was low on average and more research is needed to elucidate the exact mechanisms by which abatacept and/or glucocorticoids influences bone metabolism in patients with RA.

It is fair to acknowledge that this study has some limitations. First, it is an observational study with a relatively small sample size. This limitation may affect the generalizability of the findings and the statistical power of the analysis. Future studies with larger sample sizes are warranted to validate these results. Second, the study did not include a control group, which makes it challenging to attribute the observed changes solely to abatacept treatment. A controlled trial comparing abatacept to a placebo or an alternative treatment would provide more robust evidence regarding the effects on bone metabolism.

## Conclusions

In conclusion, our study provides some insights into the possible effects of abatacept on bone turnover markers, bone modulators, and bone health in patients with RA. The observed increase in bone formation markers suggests a potential positive effect on bone formation, highlighting the importance of addressing inflammation in optimizing bone health outcomes. The transient increase in PTH levels might raise concerns about potential bone loss in the early phases of treatment.

### Supplementary Information

Supplementary Information.

## Data Availability

Data of the analysis are available upon reasonable request to Giovanni Adami (giovanni.adami@univr.it).

## Abbreviations

RA: Rheumatoid arthritis
BMD: Bone mineral density
DMARDs: Disease modifying anti-rheumatic drugs
SvdH: Sharp van der Heijde score
BHI: Bone health index
MCI: Metacarpal index
DXA: Dual-energy X-ray absorptiometry
DAS28-CRP: Disease activity score based on 28 joints and C-reactive protein
Dkk1: Dickkopf-1
CTLA-4: Cytotoxic T lymphocyte associated antigen-4
TNF: Tumor necrosis factor
CTX: C-terminal telopeptide of type I collagen
P1NP: Procollagen I intact N-terminal peptide
B-ALP: Bone alkaline phosphatase
25OHVitD: 25OH-vitamin D
PTH: Parathyroid hormone
OPG: Osteoprotegerin
RANKL: Receptor activator of nuclear factor kappa-Β ligand
