# Supplementary material for: Changes in bone turnover markers and bone modulators during abatacept treatment
Source: Sci Rep. 2023 Oct 11;13:17183. doi: 10.1038/s41598-023-44374-2 (PMC10567677; doi:10.1038/s41598-023-44374-2)

Supplementary appendix

**Title:**  Changes in bone turnover markers and bone modulators during abatacept treatment

**Authors:** Giovanni Adami^1^, Giovanni Orsolini^1^, Maurizio Rossini^1^, Elisa Pedrollo ^1^, Anna Fratucello^2^, Angelo Fassio^1^, Ombretta Viapiana^1^, Stefano Milleri^3^, Elena Fracassi^1^, Riccardo Bixio^1^, Davide Gatti**^1^**

Supplementary figure

Figure S1. Effects of abatacept on DAS28-CRP in the study population


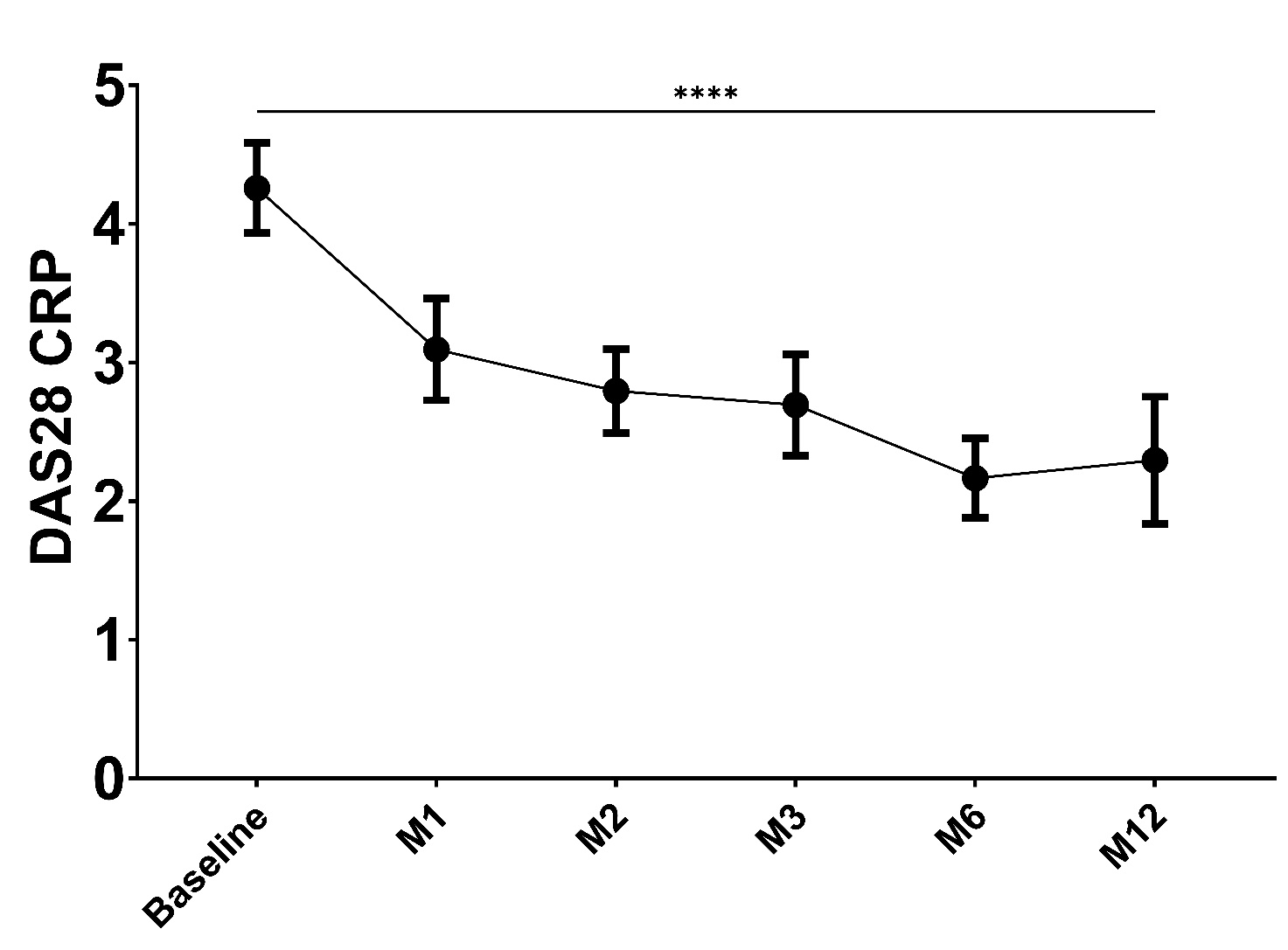


Figure S2. P1nP serum levels variations in patients taking or not taking glucocorticoids at baseline


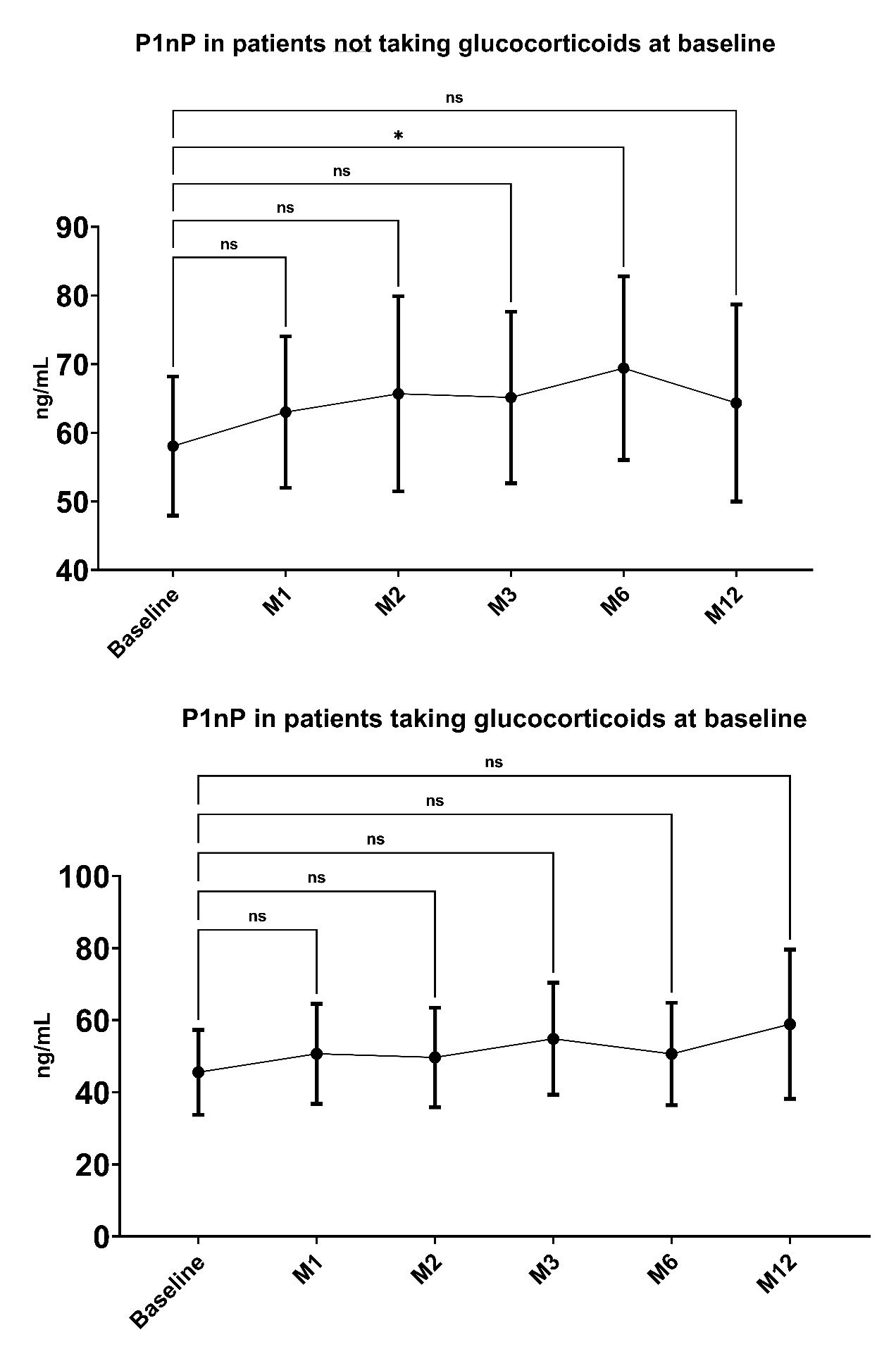


Figure S3. B-ALP serum levels variations in patients taking or not taking glucocorticoids at baseline


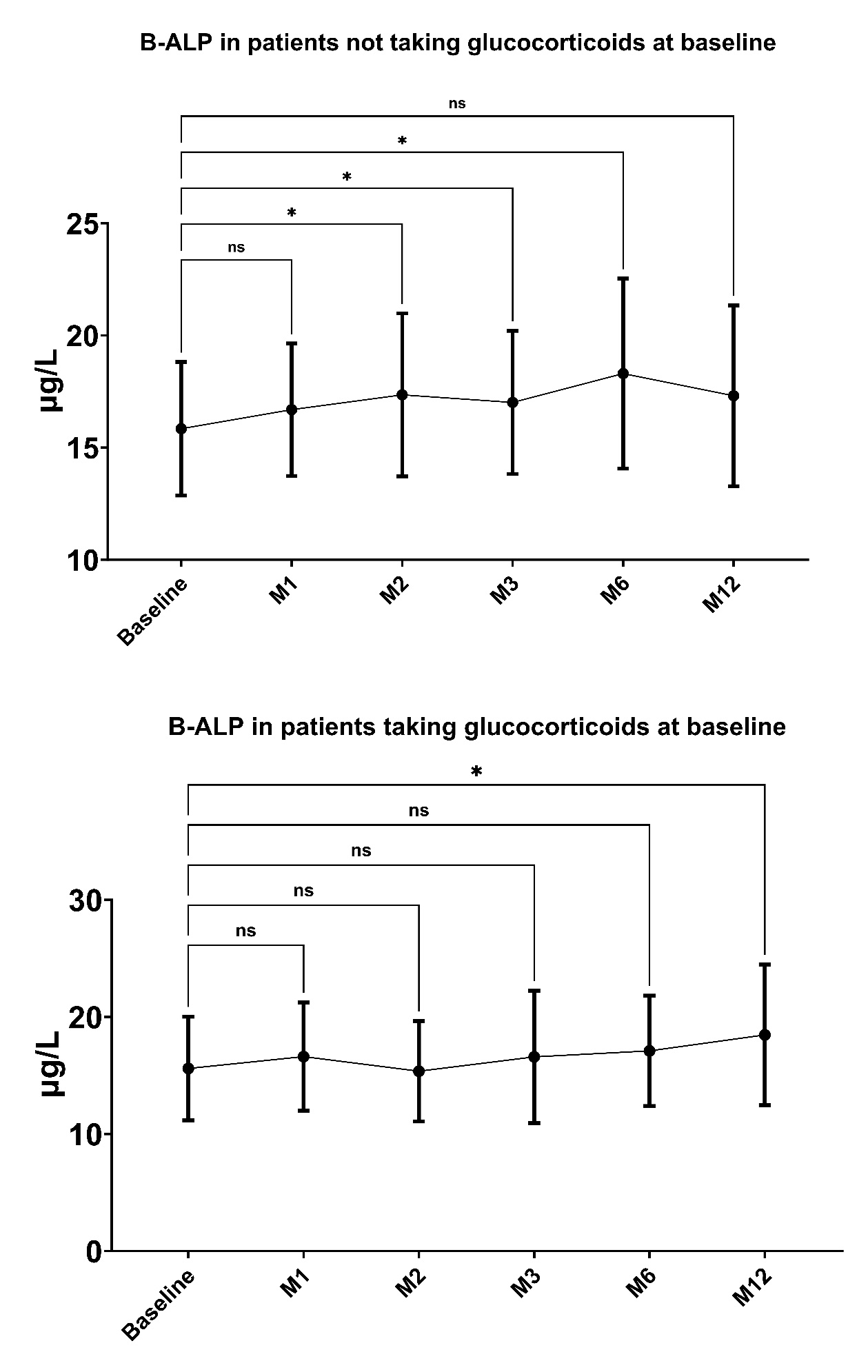


Figure S4. P1nP serum levels variations in patients with >4.0 DAS28-CRP or ≤4.0 DAS28-CRP at baseline


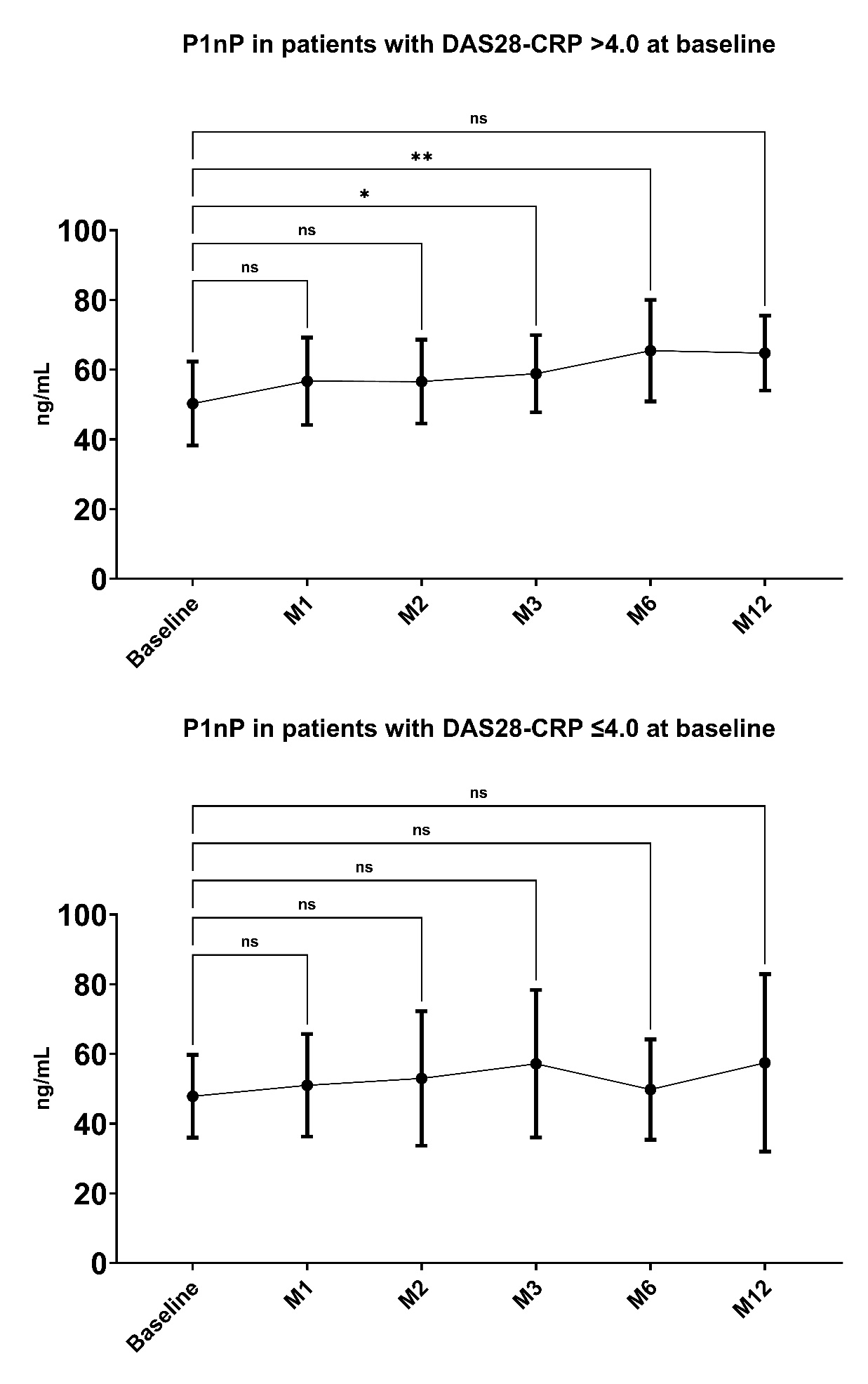

Supplement: Supplementary file 1 — Supplementary Information. [file 41598_2023_44374_MOESM1_ESM.docx]
